# A protocol for the differentiation of human embryonic stem cells into midbrain dopaminergic neurons

**DOI:** 10.1016/j.xpro.2023.102355

**Published:** 2023-06-12

**Authors:** Kaneyasu Nishimura, Emilía Sif Ásgrímsdóttir, Shanzheng Yang, Ernest Arenas

**Affiliations:** 1 Department of Medical Biochemistry and Biophysics, Karolinska Institutet, 171 77 Stockholm, Sweden; 2 Laboratory of Functional Brain Circuit Construction, Graduate School of Brain Science, Doshisha University, Kyotanabe 610-0394, Japan

**Keywords:** Developmental biology, Neuroscience, Stem Cells, Cell Differentiation

## Abstract

Here, we present a protocol for the generation of functional midbrain dopaminergic (mDA) neurons from human embryonic stem cells (hESCs), which mimics the development of the human ventral midbrain. We describe steps for hESC proliferation, induction of mDA progenitors, freezing stocks of mDA progenitors as an intermediate starting point to reduce the time to make mDA neurons, and maturation of mDA neurons. The entire protocol is feeder-free and uses chemically defined materials.

For complete details on the use and execution of this protocol, please refer to Nishimura et al. (2023).^1^

## Highlights

• Step-by-step protocol for the induction of mDA neurons from hESCs
• The protocol recapitulates key aspects of *in vivo* human midbrain development
• Frozen mDA progenitor stocks can be used for faster production of mDA neurons

## Abstract (editor highlights)

Publisher’s note: Undertaking any experimental protocol requires adherence to local institutional guidelines for laboratory safety and ethics.

## Graphical abstract

**Figure:**
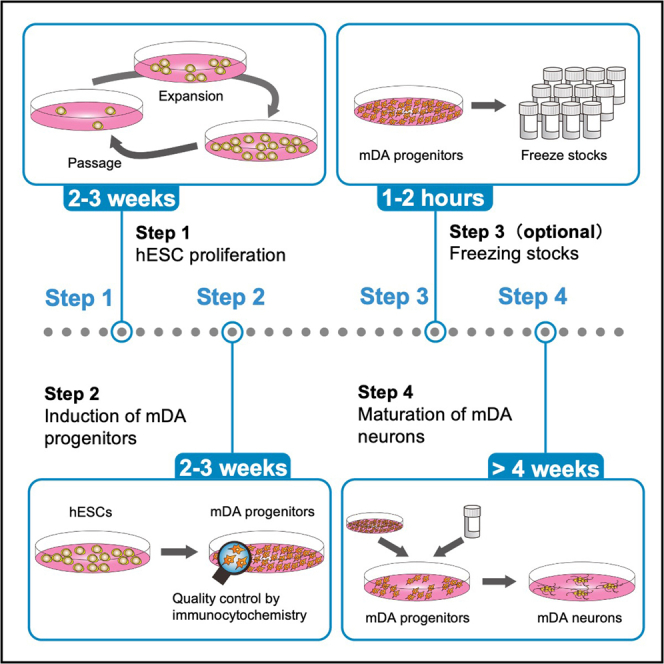

## Before you begin

Prepare all stocks and media listed in the materials and equipment section before starting cell culture. If you use reagents from alternative suppliers, you must validate them before starting the experiments. All steps should be performed under sterile conditions in a Class II biosafety cabinet. Cells should be cultivated in a humidified incubator at 37°C with 5% CO_2_.

***Note:*** Work with hESCs should be performed in compliance with local and national ethical rules, regulatory requirements, and international guidelines.

## Key resources table

**Table:** 

REAGENT or RESOURCE	SOURCE	IDENTIFIER
**Antibodies**		

Goat polyclonal anti-DCX antibody (1:500)	Santa Cruz Biotechnology	Cat#sc-8066; RRID: AB_2088494
Mouse monoclonal anti-EN1 antibody (1:50)	DSHB	Cat#4G11
Goat polyclonal anti-FOXA2 antibody (1:1,000)	R&D Systems	Cat#AF2400; RRID: AB_2294104
Rabbit polyclonal anti-LMX1 antibody (1:1,000)	Merck Millipore	Cat#AB10533; AB_10805970
Goat polyclonal anti-NGN2 antibody (1:200)	Santa Cruz Biotechnology	Cat#sc-19233; RRID: AB_477193
Rabbit polyclonal anti-NURR1 antibody (1:500)	Santa Cruz Biotechnology	Cat#sc-990; RRID: AB_2298676
Goat polyclonal anti-OTX2 antibody (1:1,000)	R&D Systems	Cat#AF1979; RRID: AB_2157172
Rabbit polyclonal anti-SOX2 antibody (1:500)	Merck Millipore	Cat#AB5603; RRID: AB_2286686
Rabbit polyclonal anti-TH antibody (1:1,000)	Merck Millipore	Cat#AB152; RRID: AB_390204
Mouse monoclonal anti-TH antibody (1:500)	ImmunoStar	Cat#22941; RRID: AB_572268
Donkey anti-Rabbit Alexa Fluor 488 secondary antibody	Thermo Fisher Scientific	Cat#A21206; RRID: AB_2535792
Donkey anti-Rabbit Alexa Fluor 555 secondary antibody	Thermo Fisher Scientific	Cat#A31572; RRID: AB_162543
Donkey anti-mouse Alexa Fluor 488 secondary antibody	Thermo Fisher Scientific	Cat#A21202; RRID: AB_141607
Donkey anti-mouse Alexa Fluor 555 secondary antibody	Thermo Fisher Scientific	Cat#A31570; RRID: AB_2536180
Donkey anti-goat Alexa Fluor 488 secondary antibody	Thermo Fisher Scientific	Cat#A11055; RRID: AB_2534102
Donkey anti-goat Alexa Fluor 555 secondary antibody	Thermo Fisher Scientific	Cat#A21432; RRID: AB_2535853

**Chemicals, peptides, and recombinant proteins**		

NutriStem hPSC XF medium	Biological Industries	Cat#05-100-1A
TeSR-E6 medium	Stem Cell Technologies	Cat#5946
Neurobasal medium	Thermo Fisher Scientific	Cat#21103049
B-27 supplement (50×)	Thermo Fisher Scientific	Cat#17504044
TrypLE Select	Thermo Fisher Scientific	Cat#12563011
L-glutamine (100×)	Thermo Fisher Scientific	Cat#A2916801
MEM non-essential amino acids (100×)	Thermo Fisher Scientific	Cat#11140-050
Penicillin/streptomycin (100×)	Thermo Fisher Scientific	Cat#10378016
STEM-CELLBANKER GMP-Grade	ZENOAQ	Cat#CB045
2-mercaptoethanol (500×)	Gibco	Cat#21985023
Defined trypsin inhibitor (DTI)	Thermo Fisher Scientific	Cat#R007100
DNase I	Sigma-Aldrich	Cat#11284932001
Human recombinant laminin-521 (LN521)	BioLamina	Cat#LN521
Human recombinant laminin-511 (LN511)	BioLamina	Cat#LN511
LDN193189	Stemgent	Cat#04-0074
Purmorphamine	Stemgent	Cat#04-0009
Y-27632	Tocris	Cat#1254
SB431542	Tocris	Cat#1614
CHIR99021	Sigma-Aldrich	Cat#SML1046
GW3965	Sigma-Aldrich	Cat#G6295
DAPT	Sigma-Aldrich	Cat#D5942
PD0325901	Sigma-Aldrich	Cat#PZ0162
SU5402	Sigma-Aldrich	Cat#SML0443
Dibutyryl-cyclic AMP (dbcAMP)	Sigma-Aldrich	Cat#D0627
Ascorbic acid	Sigma-Aldrich	Cat#A4544
Recombinant human/murine fibroblast growth factor8B (FGF8B)	PeproTech	Cat#100-25-100
Recombinant human WNT5A	R&D Systems	Cat#645-WN-010/CF
Recombinant human brain-derived neurotrophic factor (BDNF)	R&D Systems	Cat#248-BD-025/CF
Recombinant human glial cell-derived neurotrophic factor (GDNF)	R&D Systems	Cat#212-GD-050/CF
Recombinant human transforming growth factorβ3 (TGFβ3)	R&D Systems	Cat#243-B3-010/CF
DAPI	Sigma-Aldrich	Cat#D9542
Normal donkey serum	Jackson ImmunoResearch Laboratory	Cat#567-72351

**Experimental models: Cell lines**		

Human ESCs H9	Thomson et al.^2^	N/A
Human ESCs HS401	Rodin et al.^3^	N/A
Human ESCs HS975	Rodin et al.^3^	N/A
Human ESCs HS980	Rodin et al.^3^	N/A

## Materials and equipment

**Table:** 

Neural induction medium		
Reagent	Final concentration	Amount
TeSR-E6 medium	N/A	500 mL
L-glutamine	2 mM	5 mL
MEM non-essential amino acids	1×	5 mL
2-mercaptoethanol	0.1 mM	1 mL

**Table:** 

Neural differentiation medium		
Reagent	Final concentration	Amount
Neurobasal medium	N/A	500 mL
B-27 supplement	1×	10 mL
L-glutamine	2 mM	5 mL

Store at 4°C for up to 4 weeks.

***Optional:*** Penicillin/streptomycin can be supplemented in the media to prevent bacterial contamination.

***Note:*** Media can be aliquoted and stored at −20°C. Avoid repeated freezing and thawing.

**Table:** 

Storage and working concentrations of reagents			
Reagent	Stock concentration	Working concentration	Dilution factor
Y-27632	10 mM in DMSO	10 μM	1:1,000
LDN193189	500 μM in DMSO	200 nM	1:2,500
SB431542	10 mM in DMSO	10 μM	1:1,000
Purmorphamine	2 mM in DMSO	2 μM	1:1,000
CHIR99021	7.5 mM in DMSO	1.5 μM, 7.5 μM	1:5,000, 1:1,000
WNT5A	100 μg/mL in PBS(-Ca^2+^/-Mg^2+^)	100 ng/mL	1:1,000
FGF8B	100 μg/mL in PBS(-Ca^2+^/-Mg^2+^)	100 ng/mL	1:1,000
GW3965	10 mM in DMSO	10 μM	1:1,000
Ascorbic acid	100 mM in DW	200 μM	1:500
BDNF	20 μg/mL in PBS(-Ca^2+^/-Mg^2+^)	20 ng/mL	1:1,000
DAPT	10 mM in DMSO	10 μM	1:1,000
dbcAMP	500 mM in DW	500 μM	1:1,000
SU5402	5 mM in DMSO	5 μM	1:1,000
PD0325901	1 mM in DMSO	1 μM	1:1,000
GDNF	20 μg/mL in PBS(-Ca^2+^/-Mg^2+^)	10 ng/mL	1:2,000
TGFβ3	2 μg/mL in 4 mM HCl	1 ng/mL	1:2,000
DNase I	10 mg/mL	100 μg/mL	1:100

Prepare aliquots and store at −20°C. Thawed aliquots can be stored up to 4 weeks at 4°C. Recombinant proteins can be stored up to a week at 4°C. Avoid repeated freezing and thawing of the aliquots. All factors should be supplemented in the media just before use.

**Table:** 

Recipe for 10 mL of the medium (day 0)		
Reagent	Final concentration	Amount
Neural induction medium	N/A	10 mL
500 μM LDN193189	200 nM	4 μL
10 mM SB431542	10 μM	10 μL

All factors should be supplemented in the media just before use.

**Table:** 

Recipe for 10 mL of the medium (day 1 and 2)		
Reagent	Final concentration	Amount
Neural induction medium	N/A	10 mL
500 μM LDN193189	200 nM	4 μL
10 mM SB431542	10 μM	10 μL
2 mM Purmorphamine	2 μM	10 μL

All factors should be supplemented in the media just before use.

**Table:** 

Recipe for 10 mL of the medium (day 3 and 4)		
Reagent	Final concentration	Amount
Neural induction medium	N/A	10 mL
500 μM LDN193189	200 nM	4 μL
10 mM SB431542	10 μM	10 μL
2 mM Purmorphamine	2 μM	10 μL
7.5 mM CHIR99021	1.5 μM	2 μL

All factors should be supplemented in the media just before use.

**Table:** 

Recipe for 10 mL of the medium (day 5 and 6)		
Reagent	Final concentration	Amount
Neural induction medium	N/A	7.5 mL
Neural differentiation medium	N/A	2.5 mL
500 μM LDN193189	200 nM	4 μL
10 mM SB431542	10 μM	10 μL
2 mM Purmorphamine	2 μM	10 μL
7.5 mM CHIR99021	1.5 μM	2 μL

All factors should be supplemented in the media just before use.

**Table:** 

Recipe for 10 mL of the medium (day 7 and 8)		
Reagent	Final concentration	Amount
Neural induction medium	N/A	5 mL
Neural differentiation medium	N/A	5 mL
500 μM LDN193189	200 nM	4 μL
2 mM Purmorphamine	2 μM	10 μL
7.5 mM CHIR99021	1.5 μM	2 μL
100 μg/mL WNT5A	100 ng/mL	10 μL

All factors should be supplemented in the media just before use.

**Table:** 

Recipe for 10 mL of the medium (day 9 and 10)		
Reagent	Final concentration	Amount
Neural induction medium	N/A	2.5 mL
Neural differentiation medium	N/A	7.5 mL
500 μM LDN193189	200 nM	4 μL
2 mM Purmorphamine	2 μM	10 μL
7.5 mM CHIR99021	1.5 μM	2 μL
100 μg/mL WNT5A	100 ng/mL	10 μL
100 μg/mL FGF8B	100 ng/mL	10 μL

All factors should be supplemented in the media just before use.

**Table:** 

Recipe for 10 mL of the medium (day 11 to 15)		
Reagent	Final concentration	Amount
Neural differentiation medium	N/A	10 mL
2 mM Purmorphamine	2 μM	10 μL
7.5 mM CHIR99021	7.5 μM	10 μL
100 μg/mL FGF8B	100 ng/mL	10 μL

All factors should be supplemented in the media just before use.

**Table:** 

Recipe for 10 mL of the medium (day 16–20)		
Reagent	Final concentration	Amount
Neural differentiation medium	N/A	10 mL
10 μM GW3965	10 μM	10 μL
10 mM DAPT	10 μM	10 μL
100 mM ascorbic acid	200 μM	20 μL
20 μg/mL BDNF	20 ng/mL	10 μL

All factors should be supplemented in the media just before use.

**Table:** 

Recipe for 10 mL of the medium (day 21 to 27)		
Reagent	Final concentration	Amount
Neural differentiation medium	N/A	10 mL
10 mM DAPT	10 μM	10 μL
100 mM ascorbic acid	200 μM	20 μL
20 μg/mL BDNF	20 ng/mL	10 μL
5 mM SU5402	5 μM	10 μL
1 mM PD0325901	1 μM	10 μL
20 μg/mL GDNF	10 ng/mL	5 μL
2 μg/mL TGFβ3	1 ng/mL	5 μL
500 mM dbcAMP	500 μM	10 μL

All factors should be supplemented in the media just before use.

**Table:** 

Recipe for 10 mL of the medium (day 28 ∼)		
Reagent	Final concentration	Amount
Neural differentiation medium	N/A	10 mL
10 mM DAPT	10 μM	10 μL
100 mM ascorbic acid	200 μM	20 μL
20 μg/mL BDNF	20 ng/mL	10 μL
20 μg/mL GDNF	10 ng/mL	5 μL
2 μg/mL TGFβ3	1 ng/mL	5 μL
500 mM dbcAMP	500 μM	10 μL

All factors should be supplemented in the media just before use.

## Step-by-step method details

### Expansion and preparation of hESCs before differentiation of mDA progenitors

**Timing: 2–3 weeks (for all steps in this section)**

**Timing: 1 h (for step 1)**

**Timing: 6–7 days (for step 2)**

**Timing: 6–7 days (for step 3)**

**Timing: 1 h (for steps 3a to 3i)**

hESCs are cultured under xeno- and feeder-free conditions using laminin-521 (LN521), to recapitulate the embryonic stem cell niche, and are passaged at least twice before starting differentiation. Here we describe the procedure for a 6-well plate. If you use other plates, please calculate the concentration of LN521 according to the surface area (See Table 1).

***Note:*** The culture medium, PBS, TrypLE Select and DTI should be warmed in a water bath at 37°C before use.

1. Thaw hESCs.
  a. Mix 100 μL of LN521and 1,400 μL of PBS(+Ca^2+^/+Mg^2+^) (working concentration: 1 μg/cm^2^) and coat a well of a 6-well plate at 37°C for at least 2 h.
    ***Optional:*** The plate can be coated with LN521 at 4°C for overnight (15–22 h).
  b. Slowly thaw a cryovial containing approximately 500,000 cells using a water bath at 37°C until the cells are half-thawed, then immediately collect them in a 15-mL tube with 10 mL of NutriStem hPSC XF medium.
    **CRITICAL:** Do not completely thaw the cells in the water bath to avoid cell death.
  c. Centrifuge the cells at 180 G for 3–5 min at room temperature (20°C–25°C).
  d. In the meantime, wash the coated well with PBS(+Ca^2+^/+Mg^2+^) twice and add 2 mL of NutriStem hPSC XF medium supplemented with 10 μM Y-27632 to prevent cell death.
    ***Note:*** A ROCK inhibitor Y-27632 drastically improves cell viability of single-cell dissociated cells.^4^
  e. After centrifugation, discard the supernatant and resuspend the cells in 1 mL of NutriStem hPSC XF medium supplemented with 10 μM Y-27632.
  f. Seed the cells in a final volume of 3 mL of NutriStem hPSC XF medium supplemented with 10 μM Y-27632 in one well of 6-well plate.
  g. The next day, change the culture medium to 3 mL of NutriStem hPSC XF medium without Y-27632.
2. Maintenance of hESCs

**Table 1:** Coating the well

	96-Well	48-Well	24-Well	12-Well	6-Well
Surface area (cm^2^)	0.33	0.75	1.8	3.6	9.6
LN521 or LN511	5 μL	10 μL	20 μL	40 μL	100 μL
PBS(+Ca^2+^/+Mg^2+^)	70 μL	140 μL	280 μL	560 μL	1,400 μL

Maintain the hESCs in NutriStem hPSC XF medium until they reach 70–80% confluency, changing the medium daily. The volume of NutriStem hPSC XF medium should be increased from 3 mL to 8 mL according to the growth of the cells to avoid the medium turning yellow.

3. Passage of hESCs.
  a. Mix 100 μL of LN521 and 1,400 μL of PBS(+Ca^2+^/+Mg^2+^) and coat a well of a 6-well plate at 37°C for at least 2 h.
  b. Discard the supernatant of the cells in culture and wash the cells with PBS(-Ca^2+^/-Mg^2+^) twice.
  c. Add 1 mL of TrypLE Select to the well and incubate at 37°C for 4 min.
  d. Gently pipette to yield a single-cell suspension, add 1 mL of defined trypsin inhibitor (DTI) to inactivate TrypLE Select and then collect all cells in a 15-mL tube.
  e. Centrifuge the cells at 180 G for 3–5 min at room temperature (20°C–25°C).
  f. In the meantime, wash the coated well with PBS(-Ca^2+^/-Mg^2+^) twice and add 1 mL of NutriStem hPSC XF medium supplemented with 10 μM Y-27632.
  g. After centrifugation, discard the supernatant and resuspend the cells in 1 mL of NutriStem hPSC XF medium supplemented with 10 μM Y-27632.
  h. Count the cells using a hemocytometer.
  i. Seed 50,000 cells in a final volume of 2 mL of NutriStem hPSC XF medium supplemented with 10 μM Y-27632.
  j. The next day, change the culture medium to 2 mL of NutriStem hPSC XF medium without Y-27632.
  k. Change the medium daily until they reach 70–80% confluency (usually in 6–7 days).
  l. Repeat the entire process at least one more time.
    ***Note:*** We usually obtain 2-3 million cells from a well of 6-well plate when the cells reach 70%–80% confluency. But it depends on cell lines and culture conditions.
    ***Note:*** Handle carefully the plate when it contains large volumes of media to avoid spillage and contamination.
    ***Optional:*** Excess cells can be frozen and stored in liquid nitrogen.
  m. Resuspend cells dissociated in step 3 at 1 million cells/mL in STEM-CELLBANKER GMP-Grade.
  n. Distribute 0.5 mL per cryovial (500,000 cells/vial) and store at −80°C in a cold Cryo1˚C freezing container. Transfer the cryovials to a liquid nitrogen tank the next day for long-term storage.

### Differentiation of hESCs into mDA progenitors

**Timing: 3 weeks (for all steps in this section)**

**Timing: 2 days (for step 4)**

**Timing: 1 h (for steps 4a to 4i)**

**Timing: 11 days (for steps 5 to 10)**

**Timing: 1 h (for step 11)**

**Timing: 4 days (for step 12)**

**Timing: 1 h (for step 13)**

**Timing: 2 days (for step 14)**

**Figure 1:**
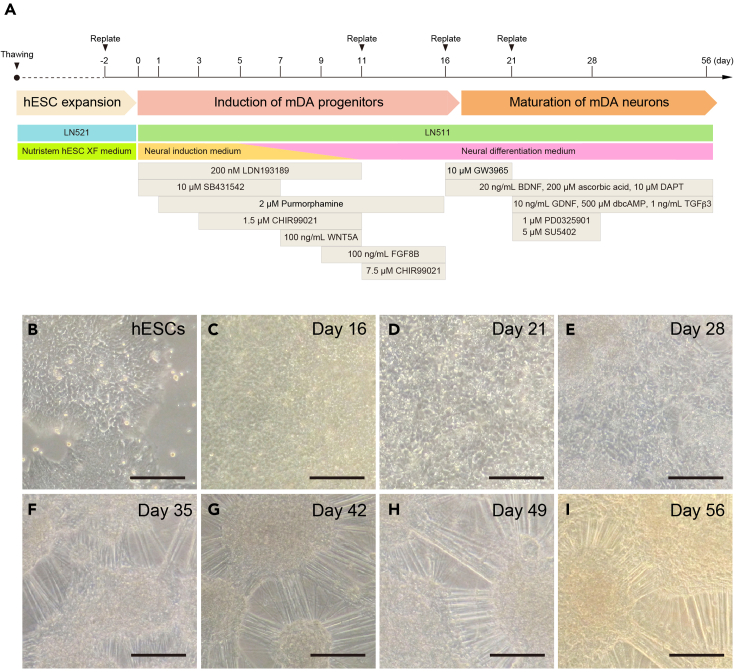
Overview of mDA neuron induction protocol from hESCs (A) A Schematic procedure for induction of mDA neurons from hESCs. (B-I) Phase contrast images during mDA differentiation of hESCs. (B) Undifferentiated hESCs, (C, D) mDA progenitor at days 16 and 21, and (E-I) mDA neuron maturation from day 28 to 56. Scale bars, 100 μm.

Differentiation of hESCs into mDA progenitors is performed in feeder-free culture conditions (Figure 1A) using LN511 to support midbrain development.^5^ Please, ensure the quality of undifferentiated hESCs before starting the differentiation. hESCs should be 70–80% confluent at the start of the differentiation and should be a homogeneous population, without any sign of differentiation such as morphological changes (fibroblast-like morphology). Here we describe the procedure for a 6-well plate.

***Note:*** Media must be changed daily using the volumes indicated as follows. If the color of the culture media turns too yellow or orange, increase the volume of media.

4. Seeding hESCs for differentiation (Day -2).
  a. Mix 100 μL of LN511 and 1,400 μL of PBS(+Ca^2+^/+Mg^2+^) and coat a well of a 6-well plate at 37°C for at least 2 h.
  b. Remove the NutriStem hPSC XF medium from the cells and wash the cells twice with PBS(-Ca^2+^/-Mg^2+^).
  c. Add 1 mL of TrypLE Select to the well and incubate at 37°C for 4 min.
  d. Gently pipette into a single cell suspension, then add 1 mL of DTI and then collect all cells in a 15-mL tube with 5 mL of NutriStem hPSC XF medium.
  e. Centrifuge the cells at 180 G for 3–5 min at room temperature (20°C–25°C).
  f. In the meantime, wash the coated well twice with PBS(-Ca^2+^/-Mg^2+^) and add 1 mL of NutriStem hPSC XF medium supplemented with 10 μM Y-27632.
  g. After centrifugation, discard the supernatant and resuspend the cells in 1 mL of NutriStem hPSC XF medium supplemented with 10 μM Y-27632.
  h. Count the cells using a hemocytometer.
  i. Seed 5 million cells in a final volume of 5 mL of NutriStem hPSC XF medium supplemented with 10 μM Y-27632, to achieve a final seeding density of 500,000 cells/cm^2^.
  j. The next day (day -1), change the culture medium: 5 mL of NutriStem hPSC XF medium without Y-27632.

**CRITICAL:** Initial cell density is particularly important for a successful differentiation outcome. A seeding density of 500,000 cells/cm^2^ should be achieved.

5. On day 0, discard the culture medium and add 5 mL of neural induction medium supplemented with 200 nM LDN193189 and 10 μM SB431542.

***Note:*** LDN193189 and SB431542 inhibit SMAD signaling to induce neuroectoderm.^6^

6. On day 1 and 2, discard the culture medium and add 5 mL of the neural induction medium supplemented with 200 nM LDN193189, 10 μM SB431542 and 2 μM purmorphamine.

***Note:*** Purmorphamine is a potent agonist of the Smoothened receptor, activates Sonic Hedgehog signaling and is used to induce floorplate identity.^7^

7. On day 3 and 4, discard the culture medium and add 5 mL of the neural induction medium supplemented with 200 nM LDN193189, 10 μM SB431542, 2 μM purmorphamine and 1.5 μM CHIR99021.

***Note:*** CHIR99021 is a GSK3β inhibitor that activates Wnt/β-catenin signaling and was used to induce caudal ventral midbrain progenitors.^8^

8. From day 5 to day11, media was gradually changed from neural induction medium to neural differentiation medium. On day 5 and 6, discard the culture medium and add a total volume 6 mL (4.5 mL of the neural induction medium + 1.5 mL) of neural differentiation medium supplemented with 200 nM LDN193189, 10 μM SB431542, 2 μM purmorphamine and 1.5 μM CHIR99021.
9. On day 7 and 8, discard the medium and add a total volume 6 mL (3 mL of neural induction medium + 3 mL of neural differentiation medium) supplemented with 200 nM LDN193189, 2 μM purmorphamine, 1.5 μM CHIR99021 and 100 ng/mL WNT5A.

***Note:*** WNT5A is a signaling molecule which activates the Wnt/Rac1 pathway and is used to promote mDA differentiation.^9^

10. On day 9 and 10, discard the culture medium and add a total volume 8 mL (2 mL of neural induction medium + 6 mL of the neural differentiation medium) supplemented with 200 nM LDN193189, 2 μM purmorphamine, 1.5 μM CHIR99021, 100 ng/mL WNT5A and 100 ng/mL FGF8B.

***Note:*** FGF8B is a growth factor that is used to induce caudal midbrain identity.^10^

11. Replate the cells on day 11.
  a. Mix 100 μL of LN511 and 1,400 μL of PBS(+Ca^2+^/+Mg^2+^) and coat a well of a 6-well plate at 37°C for at least 2 h.
  b. Remove the culture medium from the cells and wash the cells twice with PBS(-Ca^2+^/-Mg^2+^).
  c. Add 1 mL of TrypLE Select with 100 μg/mL of DNase I to the well and incubate at 37°C for 10 min.
  d. Gently pipette into a single-cell suspension, add 1 mL of DTI with 100 μg/mL of DNase I for effective cell dissociation and then collect all cells in a 15-mL tube with 5 mL of neural differentiation medium.
  e. Count the cells using a hemocytometer.
  f. Centrifuge the cells at 180 G for 3–5 min at room temperature (20°C–25°C).
  g. In the meantime, wash the coated well twice with PBS(-Ca^2+^/-Mg^2+^) and add 2 mL of the neural differentiation medium supplemented with 10 μM Y-27632, 2 μM purmorphamine, 7.5 μM CHIR99021 and 100 ng/mL FGF8B.
  h. After centrifugation, discard the supernatant and resuspend the cells in 1 mL of the neural differentiation medium supplemented with the factors in step 11g.
  i. Seed 5 million cells in a final volume of 5 mL of the neural differentiation medium supplemented with the factors in step 11g, to achieve a final seeding density of 500,000 cells/cm^2^.
12. From day 12 to day 15, discard the culture medium daily and add 5 mL of the neural differentiation medium supplemented with 2 μM purmorphamine, 7.5 μM CHIR99021 and 100 ng/mL FGF8B. Figure 1C shows an example of cell morphologies at this stage.

**Figure 2:**
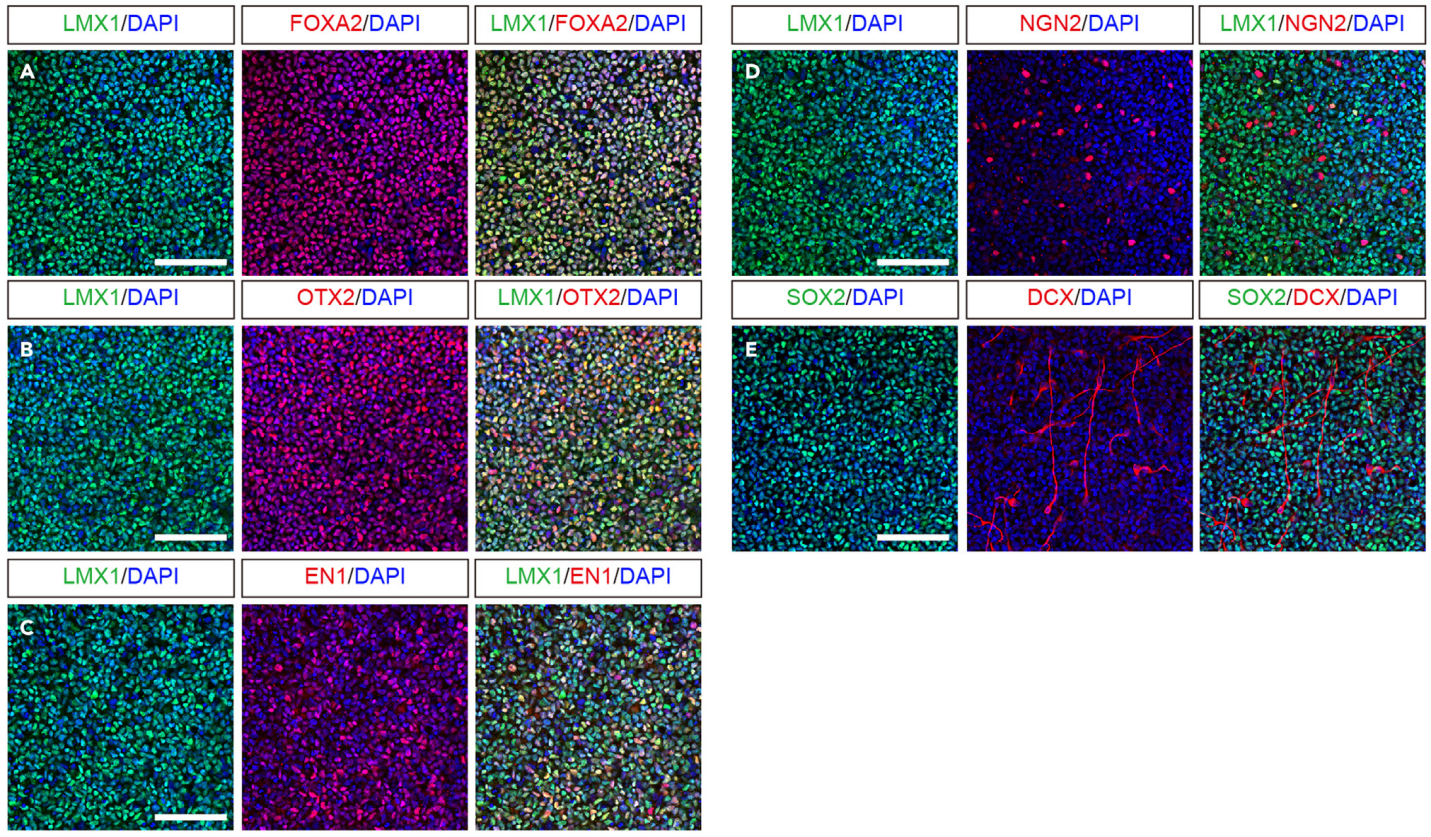
Immunofluorescence of hESC-derived mDA progenitors at day 16 (A) LMX1 (floorplate marker) and FOXA2 (ventral midbrain marker), (B) LMX1 and OTX2 (forebrain and midbrain marker), (C) LMX1 and EN1 (caudal ventral midbrain marker), (D) LMX1 and NGN2 (neurogenic maker), and (E) SOX2 (neural progenitor marker) and DCX (immature neuronal marker). On day 16, most cells are LMX1^+^, FOXA2^+^, OTX^+^2 and EN1^+^ mDA progenitors (A-C). Few cells expressed NGN2 (D) and DCX (E). Scale bars, 100 μm.
13. Replate the cells on day 16.
  a. Mix 100 μL of LN511 and 1,400 μL of PBS(+Ca^2+^/+Mg^2+^) and coat a well of a 6-well plate at 37°C for at least 2 h.
  b. Remove the culture medium from the cells and wash the cells twice with PBS(-Ca^2+^/-Mg^2+^).
  c. Add 1 mL of TrypLE Select with 100 μg/mL of DNase I to the well and incubate at 37°C for 15 min.
  d. Gently pipette into a single-cell suspension, add 1 mL of DTI with 100 μg/mL of DNase I and then collect all cells in a 15-mL tube with 5 mL of neural differentiation medium.
  e. Count the cells using a hemocytometer.
  f. Centrifuge the cells at 180 G for 3–5 min at room temperature (20°C–25°C).
  g. In meantime, wash the coated well twice with PBS(-Ca^2+^/-Mg^2+^) and add 2 mL of the neural differentiation medium supplemented with 10 μM Y-27632, 10 μM GW3965, 10 μM DAPT, 20 ng/mL BDNF and 200 μM ascorbic acid.
    ***Note:*** GW3965 is a synthetic liver X receptor ligand that is used to promote mDA neurogenesis.^11^^,^^12^ DAPT is a γ-secretase inhibitor used to promote neuronal maturation.^13^
  h. After centrifugation, discard the supernatant and resuspend the cells in 1 mL of the neural differentiation medium with the factors in step 13g.
  i. Seed 7 million cells in a final volume of 5 mL of the neural differentiation medium supplemented with the factors in step 13g, to achieve a seeding density of 700,000 cells/cm^2^.
    ***Note:*** It can be hard to completely dissociate the cells into a single-cell suspension at this time. It is normal to observe some cell clumps. We do not recommend persisted pipetting to avoid damaging the cells.
14. Quality check of the cells by immunostaining for LMX1, FOXA2, OTX2 and EN1 **(**Figure 2).
  a. Seed 200,000 cells dissociated in step 13 in a well of 96-well black flat bottom plate coated with LN511.
  b. The next day, wash the cells with PBS(-Ca^2+^/-Mg^2+^) and fix with 4% PFA for 20 min at 4°C.
  c. Remove the PFA and wash the cells three times with PBS.
  d. Remove the PBS and block the cells for 1 h at room temperature (20°C–25°C) in blocking solution (PBS with 0.1% Triton-X and 5% normal donkey serum).
  e. Dilute the primary antibodies in the blocking solution and incubate at 4°C for overnight (15–22 h). See key resources table for antibody dilution factors.
  f. Remove the antibody solution and wash the cells three times with PBS.
  g. Dilute the secondary antibodies (1:500) in the blocking solution and incubate for 1–2 h at room temperature (20°C–25°C) in the dark.
  h. Remove the secondary antibody solution and incubate with DAPI (diluted 1:5,000 in blocking solution) for 15 min at room temperature (20°C–25°C) in the dark.
  i. Remove the DAPI solution and wash the cells three times with PBS.
  j. Image the cells or store them in PBS at 4°C in the dark until imaging.
    **CRITICAL:** The concentration of CHIR99021 is essential for the cells to adopt a midbrain fate. The optimal concentration to specify mDA progenitors depends on the hESC line, the medium and the duration of CHIR99021 treatment. Therefore, the optimal concentration of CHIR99021 for midbrain patterning should be examined and adjusted for each individual cell line and culture condition before starting a full differentiation experiment. See also troubleshooting.
    As an additional guide we provide the CHIR99021 concentrations and culture medium used in other publications:
    3 μM of CHIR990211 (day 3 - day 11) in DMEM + 15% KSR (Kiriks et al., 2011).^8^
    3 μM of CHIR990211 (day 3 – day 12) in GMEM + 8% KSR (Doi et al., 2014).^14^
    0.6–0.9 μM of CHIR99021 (day 0 – day 11) in N2 and B27 medium (Kirkeby et al., 2017; Nolbrant et al., 2017).^15^^,^^16^
    0.7 μM of CHIR990211 (day 0 - day 4) + 7.5 μM of CHIR99021 (day 5 - day 9) + 3.0 μM CHIR99021 (day 10) in Neurobasal medium (Kim et al., 2021).^17^
    1.5 μM of CHIR99021 (day 3 – day 11) + 7.5 μM (day 12 – day 16) in E6 and Neurobasal medium (Nishimura et al., 2023).^1^
    ***Optional:*** The cells can be frozen and stored at this stage (day 16) in liquid nitrogen.
  k. Making an intermediate frozen stock at day 16.
    i. Resuspend the cells dissociated in step 13 at a density of 5 million cells/mL in STEM-CELLBANKER GMP-Grade.
    ii. Distribute 0.5 mL of the cell suspension per cryovial (2.5 million cells/vial) and store at −80°C in a cold Cryo1˚C freezing container. Transfer the cryovials to a liquid nitrogen tank the next day for long-term storage.
  l. Thawing the frozen stock.
    i. Mix 100 μL of LN511 and 1,400 μL of PBS(+Ca^2+^/+Mg^2+^) and coat a well of a 6-well plate at 37°C for at least 2 h.
    ii. Slowly thaw three vials (7.5 million cells in total) in a 37°C water bath, and immediately collect into a 15-mL tube with 10 mL of the neural differentiation medium.
    iii. Centrifuge the pooled cells at 180 G for 3–5 min at room temperature (20°C–25°C).
    iv. In the meantime, wash the coated well twice with PBS(-Ca^2+^/-Mg^2+^) and add 1 mL of the neural differentiation medium supplemented with 10 μM Y-27632, 10 μM GW3965, 10 μM DAPT, 20 ng/mL BDNF and 200 μM ascorbic acid.
    v. After centrifugation, discard the supernatant and resuspend in 4 mL of the neural differentiation medium supplemented with the factors in point iv.
    vi. For neuronal differentiation, seed all cells in a final volume of 5 mL of the neural differentiation medium supplemented with the factors in point iv.

### Maturation of progenitors into mDA neurons

**Timing: > 6 weeks (for all steps in this section)**

**Timing: 4 days (for step 15)**

**Timing: 6 days (for step 16)**

**Timing: > 30 days (for step 17)**

**Timing: 2 days (for step 18)**

This step is to terminally differentiate mDA progenitors into mDA neurons. Here we describe the procedure for a 6-well plate. Please choose the appropriate well for maturation culture according to the experiments you analyze.

**Figure 3:**
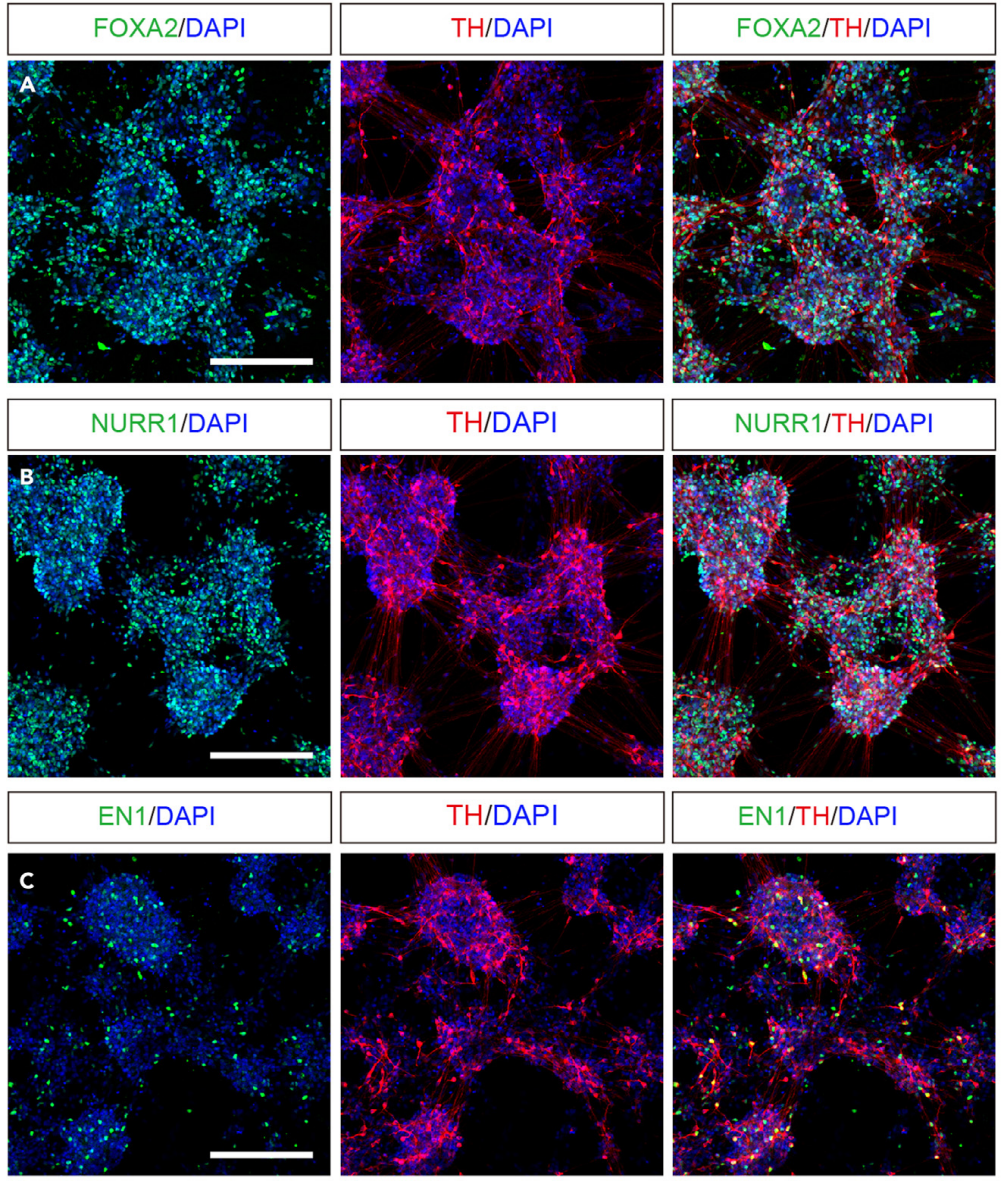
Immunofluorescence of hESC-derived mDA neurons at day 56 (A–C) (A) FOXA2 and TH (DA neuron marker), (B) NURR1 (DA neuron and post-mitotic DA neuroblast marker) and TH, (C) and EN1 and TH. Scale bars, 200 μm.

***Note:*** Media should be carefully changed (slow pipetting) after day 28 to avoid cell detachment.

***Note:*** The cells formed aggregates and neurite extensions during long-term culture (Figure 1). Action potentials and dopamine release were observed at day 56.

15. From day 17 to day 20, discard the culture medium daily and add 5 mL of neural differentiation medium supplemented with 10 μM GW3965, 10 μM DAPT, 20 ng/mL BDNF and 200 μM ascorbic acid.
  ***Optional:*** Replate the cells on day 21 to avoid clumping if the cells will be cultured beyond day 28.
  a. Mix 100 μL of LN511 and 1,400 μL of PBS(+Ca^2+^/+Mg^2+^) and coat a well of a 6-well plate at 37°C for at least 2 h.
  b. Remove the culture medium from the cells and wash the cells twice with PBS(-Ca^2+^/-Mg^2+^).
  c. Add 1 mL of TrypLE Select with 100 μg/mL of DNase I to the well and incubate at 37°C for 20 min.
  d. Gently pipette into a single-cell suspension, add 1 mL of DTI with 100 μg/mL of DNase I and then collect all cells in a 15-mL tube with 5 mL of neural differentiation medium.
  e. Count the cells using a hemocytometer.
  f. Centrifuge the cells at 180 G for 3–5 min at room temperature (20°C–25°C).
  g. In the meantime, wash the coated well twice with PBS(-Ca^2+^/-Mg^2+^) and add 2 mL of the neural differentiation medium supplemented with 10 μM Y-27632, 10 μM DAPT, 20 ng/mL BDNF, 10 ng/mL GDNF, 200 μM ascorbic acid, 500 μM dbcAMP, 1 μM PD0325901, 5 μM SU5402 and 1 ng/mL TGFβ3.
  h. After centrifugation, discard the supernatant and resuspend the cells in 1 mL of the neural differentiation medium supplemented with the factors in step 15g.
  i. Seed 5 million cells in a final volume of 5 mL of the neural differentiation medium supplemented with the factors in step 15g.
    ***Note:*** Do not replate the cells after day 22 since mature neurons are vulnerable to mechanical stress.
16. From day 22 to day 27, discard the culture medium daily and add 5 mL of neural differentiation medium supplemented with 10 μM DAPT, 20 ng/mL BDNF, 10 ng/mL GDNF, 200 μM ascorbic acid, 500 μM dbcAMP, 1 μM PD0325901, 5 μM SU5402 and 1 ng/mL TGFβ3.
17. After day 28, change half the culture medium (2.5 mL) every 2–3 days and add 2.5 mL of neural differentiation medium supplemented with 10 μM DAPT, 20 ng/mL BDNF, 10 ng/mL GDNF, 200 μM ascorbic acid, 500 μM dbcAMP and 1 ng/mL TGFβ3.
18. Check the quality of the cells by immunostaining at the desired maturation time-point (Figure 3 shows TH, FOXA2, NURR1 and EN1 at day 56). Follow procedure as in step 14, but without replating.

## Expected outcomes

This protocol leads to the generation of mDA progenitors of high quality, which can give rise to functional mDA neurons in the four hESC lines we tested. We investigated and characterized mDA progenitors and neurons by immunostaining and qPCR. The functionality of the mDA neurons was examined by HPLC, to detect DA release, and by electrophysiology. We also characterized the cell types generated by differentiation and maturation of hESCs by single-cell RNA-sequencing. We found that the *in vitro* differentiation process correctly captured human ventral midbrain development as previously defined by single-cell RNA-sequencing by La Manno et al. (2016).^18^ For more information, please refer to Nishimura et al. (2023).^1^

## Limitations

The proliferation and differentiation efficiency can vary depending on the cell line. Each cell line needs to be examined in small-scale experiments to identify any possible differentiation bias and optimize the concentration of CHIR99021 for appropriate midbrain patterning. After day 16, cells exit from cell cycle and need to be carefully manipulated (slow media changes) until the end of the experiment to avoid detachment and/or cell loss. To ensure that high quality mDA neurons are generated, the expression of midbrain transcription factors and markers must be examined as indicated above at different intermediate time points during the differentiation process. This protocol requires previous experience in working with hESCs, is time-consuming and expensive, but results in high-quality mDA neurons as defined by *in vitro* functionality and single-cell RNA-sequencing compared to the endogenous human ventral midbrain.

## Troubleshooting

### Problem 1

Cells detach during the induction of mDA progenitors (steps 4–13).

### Potential solution

Increase the volume of the culture medium. This protocol starts with high cell density (500,000 cells/cm^2^). Cells require media changes every day. Proliferation is promoted by adding CHIR99021 and/or FGF8B. This can be noticed by the fast change in color of the culture media from red towards yellow. At this stage, the culture media needs to be changed daily and increasing volumes should be used to avoid it becoming yellow.

### Problem 2

Incorrect ventral midbrain patterning of progenitors (step 14).

You need to check the cells by immunostaining for LMX1, FOXA2 and OTX2 on day16 (step 14). If you observe LMX1^+^ and FOXA2^+^ cells, and many OTX2^-^ cells, the cell identity is biased towards a caudal cell fate. Conversely, if you observe few LMX1^+^ and FOXA2^+^ and many OTX2^+^ cells, the cell identity is biased towards other rostral cell fates.

### Potential solution

The concentration of CHIR99021 needs to be adjusted. Correct CHIR99021 concentration is essential to induce ventral midbrain identity. CHIR99021 needs to be adjusted to your cell line and culture media/conditions. We recommend purchasing a large volume of CHIR99021 and making aliquots to avoid variability. A successful differentiation shows ≥ 80% of LMX1 and FOXA2 cells, ≥ 80% of LMX1 and OTX2 cells, and ≈60% of LMX1 and EN1 cells on day 16.

### Problem 3

Cells detach from the edge of well during maturation into mDA neurons (steps 15–17).

### Potential solution

Make sure that you change the culture media slowly and carefully, you have used an appropriate well and that coating is optimal (coating can be extended for overnight (15–22 h), the laminin solution may contain sodium azide, which needs to be properly washed).

### Problem 4

The number of differentiated mDA neurons is low (step 18).

### Potential solution

Mature DA neurons are sensitive to culture conditions. Lack of sufficient trophic factors or nutrients may cause the cell death and degeneration of neurites. If the color of the culture media turns yellow or orange, you may need to change it more frequently. It is important to check the proportion of mDA progenitors at day 16 since improper initial patterning will cause a low yield of mDA neurons.

### Problem 5

Difficulties dissociating the cells and cell clumping after dissociation (steps 3, 4, 11, 13 and 15).

### Potential solution

Add DNase I (1:100) in both to the TrypLE Select and to the DTI. On days 11 and 16 we incubate TrypLE Select for 15 min, potentially going up to 20 min if the day 11 cells are very hard to dissociate. For replating on day 21, increase the TrypLE Select incubation time to 20 or 30 min. We do not recommend incubation beyond 30 min to avoid cell loss.

### Problem 6

Poor attachment of the cells after plating, particularly near the edges (steps 11, 13 and 15).

### Potential solution

Coat the plates at 4°C for overnight (15–22 h) hours instead of at 37°C for 2 h. Make sure that there is enough liquid to fully cover the well. If storing the plates in the fridge, make sure that the well has not dried out.

## Resource availability

### Lead contact

Further information and requests for resources should be directed to the lead contact, Ernest Arenas, ernest.arenas@ki.se.

### Materials availability

This study did not generate any unique reagents.

### Data and code availability

This study did not generate any unique data sets or code.
